# NLRP3 Inflammasome Overactivation in Patients with Aneurysmal Subarachnoid Hemorrhage

**DOI:** 10.1007/s12975-022-01064-x

**Published:** 2022-07-11

**Authors:** Elena Díaz-García, Kapil Nanwani-Nanwani, Sara García-Tovar, Enrique Alfaro, Eduardo López-Collazo, Manuel Quintana-Díaz, Francisco García-Rio, Carolina Cubillos-Zapata

**Affiliations:** 1 Respiratory Diseases Group, Respiratory Service, La Paz University Hospital, IdiPAZ, Paseo de la Castellana 261, 28046 Madrid, Spain; 2 Biomedical Research Networking Center On Respiratory Diseases (CIBERES), Madrid, Spain; 3 Department of Intensive Care Medicine, La Paz University Hospital, Madrid, Spain; 4 The Innate Immune Response Group, La Paz University Hospital, IdiPAZ, Madrid, Spain; 5 Faculty of Medicine, Autonomous University of Madrid, Madrid, Spain

**Keywords:** Aneurysmal subarachnoid hemorrhage, NLRP3, Tissue factor, Prognosis, Vasospasm, Tako-Tsubo

## Abstract

Aneurysmal subarachnoid hemorrhage (aSAH) is an uncommon and severe subtype of stroke leading to the loss of many years of productive life. We analyzed NLRP3 activity as well as key components of the inflammasome cascade in monocytes and plasma from 28 patients with aSAH and 14 normal controls using flow cytometry, western blot, ELISA, and qPCR technologies. Our data reveal that monocytes from patients with aSAH present an overactivation of the NLRP3 inflammasome, which results in the presence of high plasma levels of interleukin (IL)-1β, IL-18, gasdermin D, and tissue factor. Although further research is needed, we propose that serum tissue factor concentration might be a useful prognosis biomarker for clinical outcome, and for Tako-Tsubo cardiomyopathy and cerebral vasospasm prediction. Remarkably, MCC-950 inhibitor effectively blocks NLRP3 activation in aSAH monocyte culture and supresses tissue factor release to the extracellular space. Finally, our findings suggest that NLRP3 activation could be due to the release of erythrocyte breakdown products to the subarachnoid space during aSAH event. These data define NLRP3 activation in monocytes from aSAH patients, indicating systemic inflammation that results in serum TF upregulation which in turns correlates with aSAH severity and might serve as a prognosis biomarker for aSAH clinical outcome and for cerebral vasospasm and Tako-Tsubo cardiomyopathy prediction.

**Supplementary Information:**

The online version contains supplementary material available at 10.1007/s12975-022-01064-x.

## Introduction

Aneurysmal subarachnoid hemorrhage (aSAH) represents a serious medical emergency with an incidence of 7.9 per 100.000 persons per year [1]. Approximately 11% of these patients die before receiving medical attention, and 40% pass away within 4 weeks after hospital admission [2]. The high mortality and morbidity of aSAH are associated with pathophysiological mechanisms including the immediate global ischemic brain injury caused by an acute increase in intracranial cerebral pressure, a decrease in cerebral blood flow, initiation of cell death signaling, blood brain barrier breakdown, and brain edema [3–5]. Also, these pathophysiological processes contribute to the appearance of complications that are closely related with the poor outcome, such as cerebral vasospasm (CVS), hydrocephalus, delayed neurological injury, and cardiopulmonary complications, such as Tako-Tsubo cardiomyopathy (TCM) [6]. Thus, efforts have been made to find prognostic markers that facilitate the detection and treatment of complications following aSAH [7]. Some studies point to inflammation as the main mechanism underlying aSAH complications [8, 9]. Recently, increasing evidence has indicated the role of the NLRP3 (nucleotide binding and oligomerization domain-like receptor family pyrin domain-containing 3) inflammasome as a key component of post-SAH inflammatory response in animal models [10–21]. In fact, activation of NLRP3 inflammasome in pathology of intracerebral hemorrhage has been recently reviewed [22]. However, to our knowledge, there is paucity of human studies confirming NLRP3 overactivation in aSAH patients.

Growing evidence suggests that the inflammasome might be an upstream target that controls “sterile” inflammation pathways involved in several inflammatory disorders [23]. Briefly, NLRP3 recruits caspase-1 (CASP1) in the presence of ASC (apoptosis-associated speck-like protein containing a CARD), resulting in the cleavage of pro-inflammatory cytokines: interleukin (IL)-1β and IL-18 [24, 25]. Also, CASP1 cleaves gasdermin D (GSDMD) to induce pyroptosis, which is inflammatory programmed cell death accompanied by increased plasma membrane permeability [26]. Moreover, tissue factor (TF), an essential initiator of coagulation cascades involved in the pathophysiology of several diseases [27], is released from pyroptotic macrophages [28]. Interestingly, patients with aSAH present increased levels of TF in cerebrospinal fluid which was related to CVS development [29–32], suggesting a possible role of TF in aSAH severity. However, the expression of serum TF has not yet been assessed in aSAH patients.

Understanding the role of NLRP3 inflammasome in aSAH human patients could be of great significance for the treatment of this disease and the prevention of its associated complications. Thus, in this study, we aimed to assess the expression of inflammasome components as well as their association with aSAH clinical indicators, complications, and outcome.

## Methods

### Patients and Healthy Participants

Twenty-eight patients who fulfilled the diagnostic criteria for aSAH according to the European Stroke Organization guidelines for the management of intracranial aneurysms and subarachnoid hemorrhage were recruited at La Paz University Hospital, Madrid, Spain. The study was conducted in accordance with the ethical guidelines of the 1975 Declaration of Helsinki and was approved by the Committee for Human Subjects of La Paz University Hospital (PI-3405). All the participants provided written consent for the study. Patient management was carried out in accordance with the European guidelines for the management of aSAH (ESO) [33]. Patient functional outcome was performed 6 months after discharge from the ICU; Glasgow Outcome Scale Extended (GOS-E) was recorded by telephone call. Fourteen sex- and age-matched normal controls (NC) with no history of aSAH or any other significant illness (including patients with a past medical history of intracranial hemorrhage, head trauma, or any respiratory, oncologic, or systemic inflammatory disease) were also recruited.

Monitoring of complications was performed during intensive care unit (ICU) stay using serial computed tomography (CT) (hydrocephalus, delayed neurological injury), echography (TCM), daily clinical and neurological evaluation (CVS, delayed neurological injury, hydrocephalus, TCM), daily transcranial Doppler (CVS, hydrocephalus), and computed tomography angiography (CTA) and/or therapeutic arteriography if appropriate (CVS) in addition to advanced ICU monitoring (invasive blood pressure, hourly diuresis, etc.). CVS was defined as a Lindergaard index ≥ 3 (mean MCA velocity ≥ 120 cm/s), performing CT angiography and/or diagnostic and/or therapeutic arteriography in doubtful and/or persistent cases despite initial management [34]. Delayed neurological injury was defined as the occurrence of focal neurological impairment (hemiparesis, aphasia, apraxia, hemianopsia, or neglect), or a decrease of at least 2 points on the Glasgow Coma Scale (GCS), lasting for at least 1 h (not evident immediately after of the occlusion of the aneurysm nor attributable to other causes that can be ruled out by clinical, analytical, or imaging tests) and/or the appearance of cerebral infarcts in the imaging tests in the first 6 weeks after aSAH that were not present in imaging tests 24–48 h after aneurysm occlusion not attributable to the surgical or endovascular process. TCM was defined by the presence of transient left ventricular dysfunction presenting as apical ballooning or midventricular, basal, or focal wall motion abnormalities. Chronic hydrocephalus was defined as hydrocephalus persisting over time (> 2–3 weeks) despite closing the external ventricular drain.

The study was conducted in accordance with the ethical guidelines of the 1975 Declaration of Helsinki and was approved by the Committee for Human Subjects of La Paz University Hospital (PI-3405). All the participants provided written consent for the study; this consent was signed by the patient or NC or by a relative or representative in case the patient does not have the capacity to sign the consent. Patient management was carried out in accordance with the European guidelines for the management of aSAH (ESO) [33]. In addition, perioperative treatment of CVS was carried out inducing hypertension with noradrenaline, and following clinical and Doppler. When CVS persists, patients were referred to interventional radiology for intra-arterial nimodipine treatment. Finally, treatment of acute hydrocephalus was the placement of an external ventricular drain. The monitored complications are known entities that are usually sought and monitored during aSAH, so all patients were diagnosed and treated following identical clinical patterns.

### Serum Isolation and Analysis

Blood samples were collected by venipuncture into 3-mL serum-separating tubes on day 1 (*n* = 28) (0–24 h), day 2–5 (*n* = 24) (48–120 h), and on day 7–10 (168–240 h) (*n* = 24) after aSAH event. Patient follow-up was not possible in patients who died (*n* = 4). NC were sampled at only one-time point and the sampling and processing were identical for every group. These tubes were centrifuged at 3000 rpm during 5 min for serum collection. Serum was then aliquoted and stored at − 80 °C for further analysis. Specific enzyme-linked immunosorbent assay (ELISA) kits were used for the measurement of IL-18, GSDMD, and TF according to manufactures’ instructions as available in Table e2, Epoch 2 Microplate Spectrophotometer reader was used (BioTek). Inflammatory cytokine IL-1β concentration was measured using BD Human Inflammatory Cytokine CBA kit (551,811, Becton–Dickinson Biosciences), acquired by BD FACS-Calibur flow cytometer (Becton–Dickinson Biosciences) and analyzed by FCAP Array software (Becton–Dickinson Biosciences).

### Monocytes Culture

Eighteen milliliters of blood was collected by venipuncture into EDTA (ethylenediamine tetraacetic acid) tubes on day 1 (*n* = 28) (0–24 h), day 2–5 (*n* = 18) (48–120 h), and on day 7–10 (168–240 h) (*n* = 18) after aSAH event. Patient follow-up was not possible in patients who died (*n* = 4) and in those in which high quantities of blood extraction were no longer recommended (*n* = 6). NC were sampled at only one-time point and the sampling and processing were identical for every group. Blood samples were layered on top of 10-mL Ficoll-Paque Plus (Amersham Biosciences) and centrifuged at 1500 rpm for 20 min at 24 °C. PBMCs (peripheral blood mononuclear cells) were removed from the interphase and washed two times in PBS (phosphate-buffered saline). Cells were then resuspended in Roswell Park Memorial Institute (RPMI) 1640 medium supplemented with 100 U/mL penicillin and 100 μg/mL streptomycin. A total of 0.5 × 10^6^ monocytes per well (6-well plates) were seeded and enriched by adherence for 1 h in media culture without fetal bovine serum. The medium was then replaced with fresh culture media supplemented with 10% fetal bovine serum. Cells were incubated at 37 °C and 5% CO_2_ for 16 h and then were collected for further analysis.

### Flow Cytometry

Monocytes from aSAH patients at day 1 (*n* = 28) (0–24 h), day 2–5 (*n* = 18) (48–120 h), and day 7–10 (168–240 h) (*n* = 18) after aSAH event and from NC (*n* = 14) were washed two times with PBS and then treated following a standard protocol using the Transcription Factor Buffer Set (Becton–Dickinson Biosciences). Cells were labeled (30 min, 4 °C) with the anti-CD14, anti-NLRP3, and anti-ASC antibodies detailed in Table e1. Additionally, caspase-1 activity was detected using the FAM FLICA caspase-1 kit following the manufacturer’s protocol (Bio-Rad Laboratories, Inc.). Cells were acquired using BD FACS-Calibur flow cytometer (Becton–Dickinson Biosciences), and data were analyzed using FlowJo vX.0.7 software (FlowJo). Gating schemes are showed at Fig. e1.

### Immunoblots

Total cell extracts were prepared from monocytes of aSAH patients at day 1 (*n* = 6) and NC (*n* = 3) using RIPA buffer supplemented with protease and phosphatase inhibitors (ThermoFisher Scientific). Equal amounts of protein from each sample were separated by SDS-PAGE (sodium dodecyl-sulfate polyacrylamide gel electrophoresis) and blotted onto iBlot Gel Transfer Stacks (Invitrogen). These nitrocellulose membranes were then probed with anti-NLRP3 mouse mAb (119–14,885; Ray Biotech), anti-caspase-1 mouse mAb (MAB6215; R&D Systems), and anti-β actin antibody (Abcam, UK) to control for protein loading, followed by a horseradish peroxidase (HRP)-conjugated secondary anti-mouse (Cell Signaling, UK). Antibody binding was detected by enhanced chemiluminescence (ECL) (Amersham-Pharmacia-Biotech, UK).

### mRNA Isolation and Quantification by qPCR

RNA was extracted using High Pure RNA Isolation Kit (Roche Diagnostics, Switzerland) from aSAH patients at day 1 (*n* = 28) and NC (*n* = 14) monocytes. One microgram of RNA was retrotranscribed using High-Capacity cDNA Reverse Transcription kit (Applied Biosystems, USA). RNA levels were measured by qPCR using QuantiMix Easy kit (Biotools, Spain) and Light-Cycler system (Roche Diagnostics, Switzerland) and results normalized to 18S expression. Primer sequences are listed in Table e3 and were synthesized by Eurofins Scientific SE (Luxembourg).

### NLRP3 Inhibition Assays

Monocytes from randomly selected aSAH patients at day 1 (*n* = 5) and NC (*n* = 4) obtained as described above were used for NLRP3 inhibition assays. A total of 0.5 × 10^6^ monocytes per well were cultured in M6 plates and treated or not with 5 μM MCC-950 (INH-MCC, Ibian Technologies S.L). Cells were incubated at 37 °C and 5% CO_2_ for 16 h and then both monocytes and supernatants were collected for further analysis. Monocytes were washed two times with PBS and then treated following a standard protocol using the Transcription Factor Buffer Set (Becton–Dickinson Biosciences). Cells were labeled (30 min, 4 °C) with the anti-CD14, anti-NLRP3, and anti-ASC antibodies detailed in Table e1. Additionally, caspase-1 activity was detected using the FAM FLICA caspase-1 kit following the manufacturer’s protocol (Bio-Rad Laboratories, Inc.). Cells were acquired using BD FACS-Calibur flow cytometer (Becton–Dickinson Biosciences), and data were analyzed using FlowJo vX.0.7 software (FlowJo). Gating schemes are showed at Fig. e1. Supernatants were stored at − 80 °C until analysis by ELISA (IL-18, GSDMD and TF) according to manufactures’ instructions as available in Table e2 or BD Human Inflammatory Cytokine CBA kit (IL-1β), as previously described.

### Erythrocyte Lysate Stimulation Models

Monocytes from healthy volunteers were obtained as previously described. 0.5 × 10^6^ monocytes per well were cultured in M6 plates. First, cells were pre-treated or not with 5 µM MCC-950 (INH-MCC, IbianTechnologies S.L., Spain) for 1 h. Then, without washing the inhibitor, cells were stimulated with various concentrations (0%, 10%, 20%, 50%) of their own erythrocyte lysate for 16 h. To prepare the erythrocyte lysate, the erythrocytes from each healthy volunteer were obtained from the lower phase after Ficoll-Paque Plus centrifugation. Then erythrocytes were treated with 1:1 H_2_O and submitted to 5 freeze–thaw cycles. Cells were incubated at 37 °C and 5% CO_2_ for 16 h and then both monocytes and supernatants were collected for further analysis. Monocytes were washed two times with PBS and then treated following a standard protocol using the Transcription Factor Buffer Set (Becton–Dickinson Biosciences). Cells were labeled (30 min, 4 °C) with the anti-CD14, anti-NLRP3, and anti-ASC antibodies detailed antibodies detailed in Table e1. Additionally, caspase-1 activity was detected using the FAM FLICA caspase-1 kit following the manufacturer’s protocol (Bio-Rad Laboratories, Inc.). Cells were acquired using BD FACS-Calibur flow cytometer (Becton–Dickinson Biosciences), and data were analyzed using FlowJo vX.0.7 software (FlowJo). Gating schemes are showed at Fig. e1. Supernatants were stored at − 80 °C until analysis by ELISA (IL-18, GSDMD and TF) according to manufactures’ instructions as available in Table e2 or BD Human Inflammatory Cytokine CBA kit (IL-1β), as previously described.

### Statistical Analyses

Data are presented as mean ± standard error mean (SEM) unled otherwise stated. Data distribution was assessed using Anderson–Darling and D’Agostino-Pearson tests for normal distribution. Comparisons between the NC subjects’ and aSAH patients’ groups were performed using nonparametric Mann–Whitney 2-tailed test. Comparisons among aSAH patients at different time points were performed by one-way ANOVA (analysis of variance) with Bonferroni’s correction for multiple comparisons. The analysis of TF levels association with outcomes was performed by two-way ANOVA with Bonferroni’s correction for multiple comparison or unpaired *t*-test. For the in vitro and ex vivo studies, we employed two-way ANOVA with Bonferroni’s correction for multiple comparisons. Correlations were assessed with Spearman’s test. *P* < 0.05 was considered significant.

## Results

### Characteristics of the Study Subjects

aSAH patients (*n* = 28) and normal controls (NC) were homogeneous in sex (71% and 76% females, respectively) and age (56 ± 12 vs 55 ± 14 years, respectively). Detailed clinical characteristics of patients with aSAH are shown in Table 1. Regarding complications frequencies, nine patients (32.1%) developed cerebral vasospasm and, 14 (50%) acute and 13 (46.4%) chronic hydrocephalus. Moreover, six patients presented TCM (21.4%) and four patients (14.2%) died in the 90-day follow-up period.

**Table 1:** Demographics, medical history, admission data, and prognostic scales of patients with aneurismal subarachnoid hemorrhage

Baseline characteristics	*n* = 28
Age (years)	56 ± 12
Women	20 (71%)
Race:	
White	24 (86%)
Asian	2 (7%)
Black	1 (4%)
Hispanic	1 (4%)
Prognostic scales:	
APACHE II	11.1 ± 8.3
SOFA	5 ± 3.8
WFNS	2 (IQR 1–4)
Glasgow Coma Scale	11.7 ± 4.2
Eyes	2.9 ± 1
Verbal	3.8 ± 1.5
Motor	5.3 ± 1.5
Complications	
Cerebral vasospasm (CVS)	9 (32.1%)
Acute hydrocephalus,	14 (50%)
Chronic hydrocephalus,	13 (46.4%)
Delayed neurological injury	6 (21.4%)
Tako-Tsubo cardiomyopathy (TCM)	6 (21.4%)

*IQR*, interquartile range; *WFNS*, World Federation of Neurosurgical Societies. Data are presented as *n* (%), mean ± standard deviation or median (interquartile range) according their type and distribution

### Patients with aSAH Exhibit NLRP3 Inflammasome Activation

**Fig. 1:**
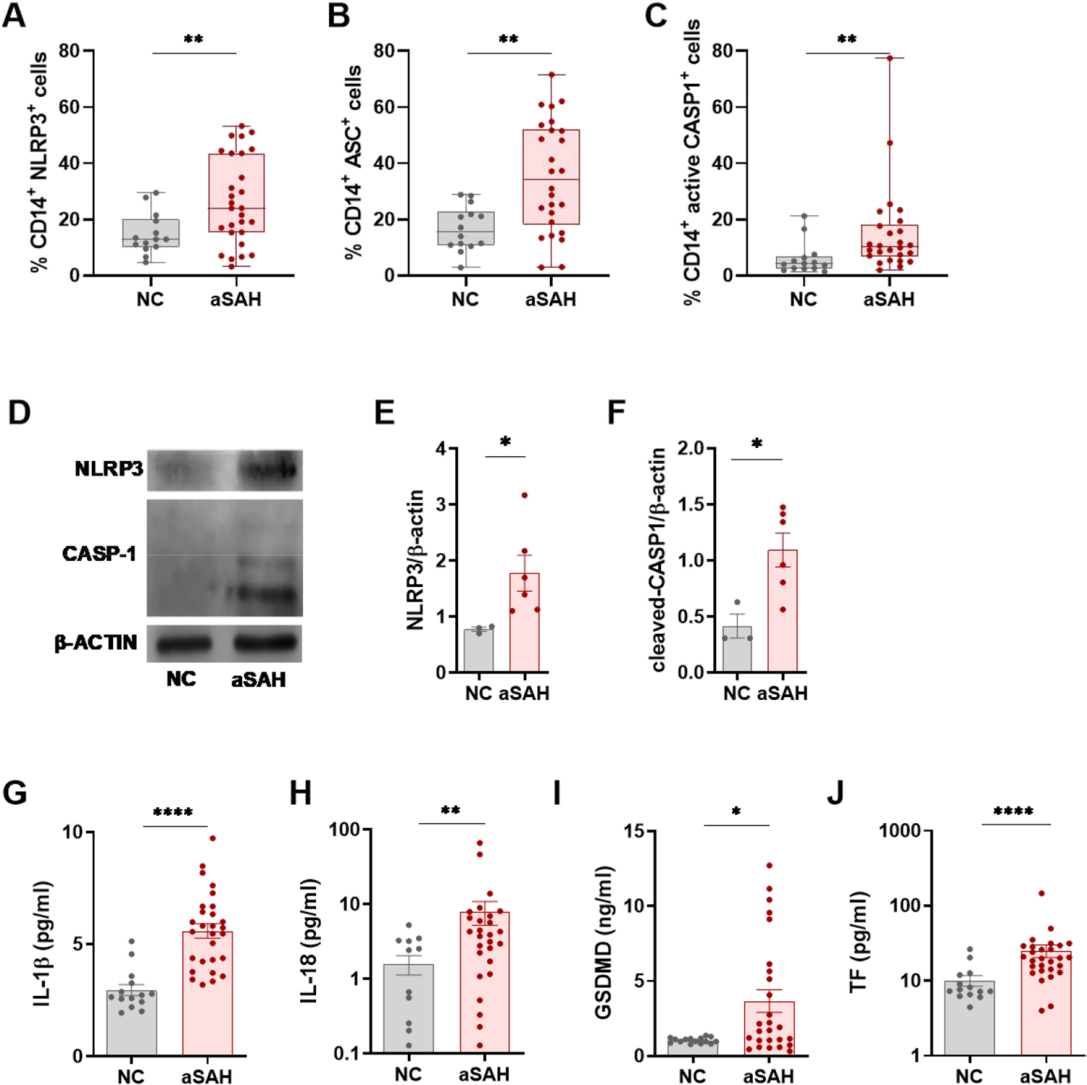
NLRP3 inflammasome activation is increased in patients with aSAH. **A**–**C**, NLRP3 (**A**), ASC (**B**), and active CASP1 (**C**) expression in monocytes from NC (*n* = 14) and patients with aSAH (*n* = 28) determined by flow cytometry. **D**–**F**, Representative western blot (**D**), quantification for NLRP3 (**E**), and active-CASP1 (**F**) in monocytes from CS (*n* = 3) and patients with aSAH (*n* = 6). **G**–**H**, IL-1β (**G**), IL-18 (**H**) determined by CBA and enzyme-linked immunosorbent assay (ELISA) respectively in serum from CS (*n* = 14) and patients with aSAH (*n* = 28). **I–J**, GSDMD (**I**) and TF (**J**) protein concentration determined enzyme-linked immunosorbent assay (ELISA) in serum from CS (*n* = 14) and patients with aSAH (*n* = 28). Comparisons between groups were performed by unpaired t test. Mean ± SEM is shown. *P < 0.05, **P < 0.01, ****P < 0.0001

As a first approach, we analyzed inflammasome component expression by flow cytometry in monocytes from patients with aSAH and NC. Our data reveal a significant increase in NLRP3, ASC, and active CASP1 intracellular expression in monocytes from patients with aSAH compared with NC (Fig. 1A−C, Fig. e2A). To further corroborate these findings, we analyzed inflammasome components by western blot in monocytes’ lysates. Similarly, we found higher expression of both NLRP3 and active CASP1, (Fig. 1D−F). In addition, serum of aSAH patients exhibited higher levels of the inflammasome end-products: IL-1β, IL-18, GSDMD, and TF (Fig. 1G−J, Fig. e2B−E). Notably, the mRNA levels of both components and products of the inflammasome were also upregulated in patients with aSAH (Fig. e2F). Altogether, these data suggest an over-activity of inflammasome complex in aSAH monocytes.

### Tissue Factor Serum Concentration Increases with aSAH Severity

**Fig. 2:**
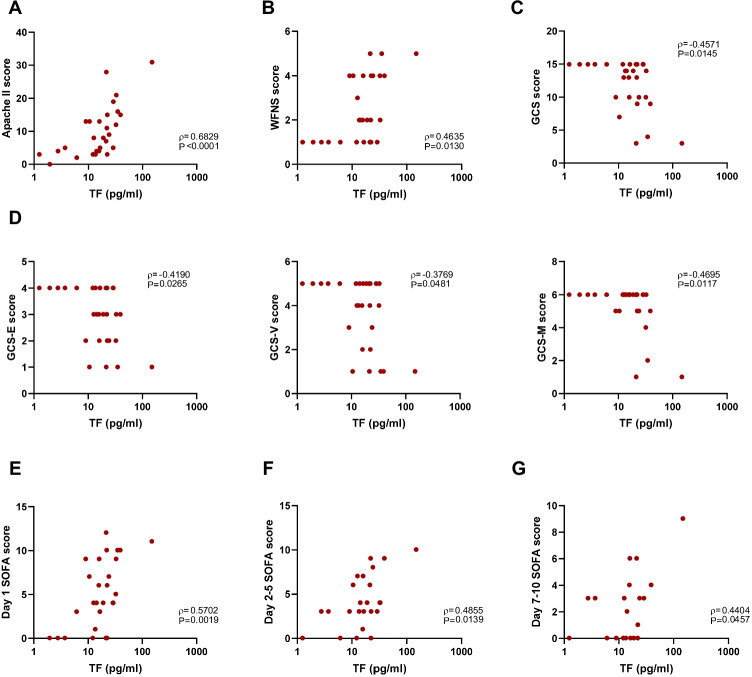
Tissue factor serum levels correlated with aSAH severity. **A–D** Correlation between TF protein concentration determined by ELISA in serum from aSAH patients and Acute Physiology and Chronic Health Disease Classification System II (Apache II) score (*n* = 28) (**A**), World Federation of Neurological Surgeons (WFNS) score (*n* = 28) (**B**), Glasgow coma scale (GCS) score (*n* = 28) (**C**), and its components GCS-eye score (GCS-E, left panel), GCS-verbal score (GCS-V middle panel) and GCS-motor score (GCS-M, right panel) (**D**). **E–G** Sequential Organ Failure Assessment (SOFA) score at day 1 (*n* = 28) (**E**), day 2–5 (*n* = 24), (**F**) and day 7–10 (*n* = 21) (**G**). Spearman’s correlation coefficients (ρ) and *P*-values are shown

We then explored the potential implication of inflammasome activation in aSAH severity. To do so, we assessed the correlation of inflammasome serum soluble products (IL-1β, IL-18, GSDMD, and TF) with clinical severity scales. Our data showed that TF serum levels positively correlated with severity and prognostic scales such as Acute Physiology and Chronic Health Disease Classification System II (Apache II), and World Federation of Neurological Surgeons (WFNS) scores (Fig. 2A−B). Furthermore, TF serum levels inversely correlated with Glasgow Coma Scale (GCS) score (Fig. 2C). In fact, TF negatively correlates with each one of the three GCS score components: eyes (GCS-E), verbal (GCS-V), and motor (GCS-M) scores (Fig. 2D). Moreover, TF serum levels positively correlated with severity scale Sequential Organ Failure Assessment (SOFA) either measured at day 1, day 2–5, or day 7–10. Meanwhile IL-1β, IL-18, and GSDMD do not show significant correlations with the scales explored (Table e4). These findings suggested the importance of TF in aSAH pathophysiology and prompted us to explore the possible role of TF as a biomarker for aSAH complications and prognosis.

### Serum Tissue Factor Correlated with aSAH Functional Outcome

**Fig. 3:**
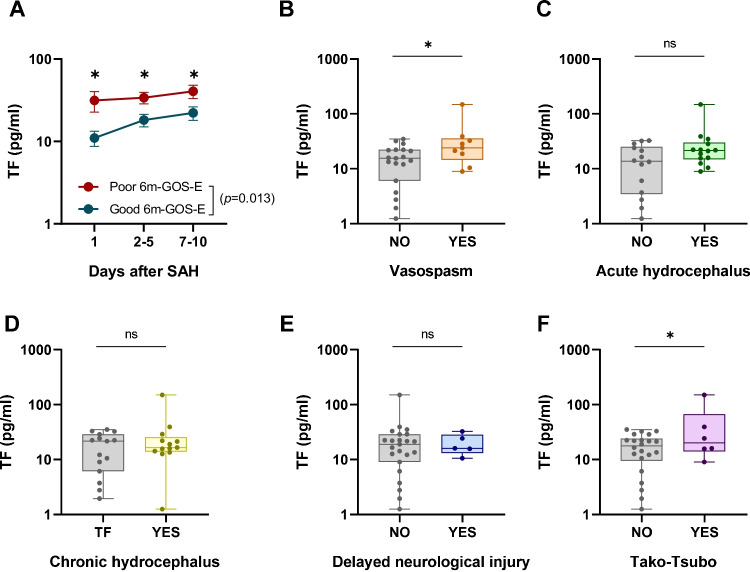
Tissue factor is related with aSAH clinical outcome and CVS and TCM development. **A** Tissue factor serum levels over time in patients with aSAH that develop poor outcome (Glasgow Outcome Scale Extended (GOS-E) < 8 at 6 months post –ICU discharge, red line, *n* = 13) or good outcome (GOS-E = 8 at 6 months post –ICU discharge, blue line, *n* = 15). Comparisons between groups were performed by unpaired two-way ANOVA with Bonferroni’s multiple comparison. Mean ± SEM is shown. *P < 0.05. **B** TF protein concentration determined by ELISA in serum from aSAH patients with (YES, *n* = 19) or without (NO, *n* = 9) vasospasm. **C** TF protein concentration determined by ELISA in serum from aSAH patients with (YES, *n* = 14) or without (NO, *n* = 14) acute hydrocephalus. **D** TF protein concentration determined by ELISA in serum from aSAH patients with (YES, *n* = 15) or without (NO, *n* = 13) chronic hydrocephalus. **E** TF protein concentration determined by ELISA in serum from aSAH patients with (YES, *n* = 23) or without (NO, *n* = 5) delayed neurological injury. **F** TF protein concentration determined by ELISA in serum from aSAH patients with (YES, *n* = 22) or without (NO, *n* = 6) Tako-Tsubo. Comparisons between groups were performed by unpaired *t* test. Mean ± SEM is shown. *P < 0.05

To accurately assess TF implication in aSAH prognosis, we recorded patient functional outcome 6-month after ICU discharge by Glasgow Outcome Scale-Extended (GOS-E). Strikingly, TF serum levels over time were significantly different between the good outcome group (GOS-E = 8) and poor outcome (GOS-E < 8) group. In this line, we explored TF serum levels in patients with and without frequent aSAH complications such as cerebral CVS, acute and chronic hydrocephalus, delayed neurological injury, and TCM. Our data revealed that TF serum concentrations at the admission day were higher in patients who developed CVS or TCM, while no differences were found for the other complications assessed (acute and chronic hydrocephalus and delayed neurological injury) (Fig. 3A−E). Interestingly, TF positively correlated with D-dimer and troponin levels (Fig. e3A and B), which have been previously suggested as indicators of CVS and TCM risk respectively [35, 36]. Although more evidence is needed to confirm TF role as a prognostic biomarker for aSAH complications, this data sow the seeds for further investigations in this context.

### MCC-950 Reduces Inflammasome-Mediated Tissue Factor-Release

**Fig. 4:**
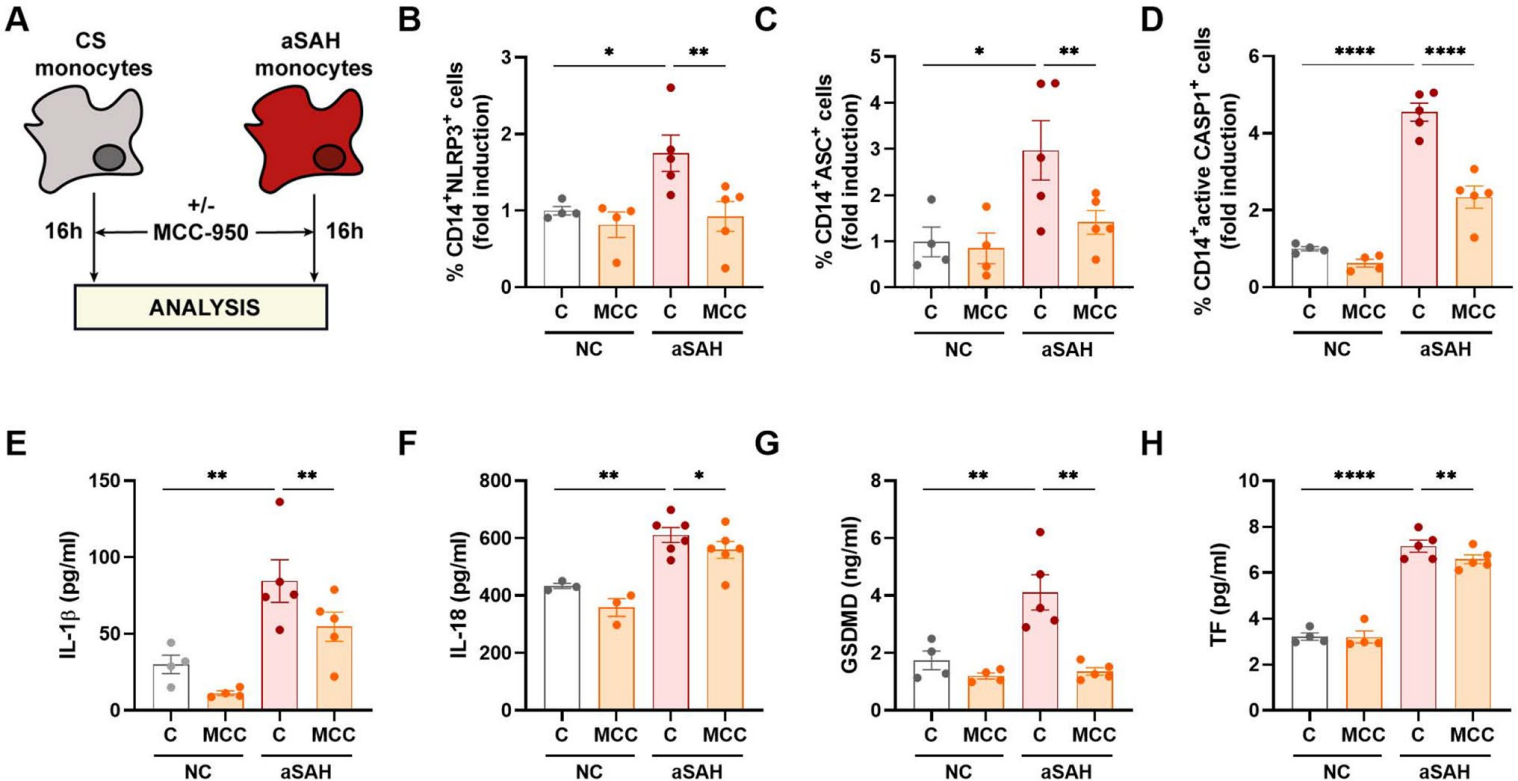
MCC-950 effectively blocks NLRP3 inflammasome activation in aSAH monocytes. **A** Schematic representation of ex vivo experiments. **B–D** NLRP3 (**B**), ASC (**C**), and active CASP1 (**C**), expression in monocytes from CS (*n* = 4) and patients with aSAH (*n* = 5) determined by flow cytometry. **E–H** IL-1β (**E**), IL-18 (**F**), GSDMD (**G**), and TF (**H**) protein concentration determined by CBA or ELISA in supernatants from CS (*n* = 4) and patients with aSAH (*n* = 5) treated or not with MCC-950. Comparisons between groups were performed by two-way ANOVA with Bonferroni’s multiple comparison. Mean ± SEM is shown. *P < 0.05, **P < 0.01, ****P < 0.0001

We further evaluated the potential role of NLRP3 inflammasome activation in TF release to extracellular space in aSAH patients. First, we assessed the correlation between TF serum levels and NLRP3 expression in monocytes. Our data show that TF serum levels increased along NLRP3 expression in monocytes suggesting a possible role of inflammasome in increased TF serum levels observed in aSAH patients (Fig. e3C). To corroborate, we treated aSAH and NC monocytes with the NLRP3 inhibitor MCC-950 (Fig. 4A). Our data showed that MCC-950 treatment effectively reduced NLRP3 and ASC protein expression and CASP1 activation in aSAH monocytes (Fig. 4B−D). Furthermore, the supernatant protein analysis reveal that IL-1β, IL-18, GSDMD, and TF significantly decreased after MCC-950 treatment (Fig. 4E−H). This data indicates NLRP3 inflammasome activation could underlie TF overexpression in aSAH serum, highlighting NLRP3 inflammasome inhibitors as a potential therapy for aSAH and its related complications such as CVS and TCM.

### Erythrocyte Lysate Activates NLRP3 Inflammasome in Monocytes

**Fig. 5:**
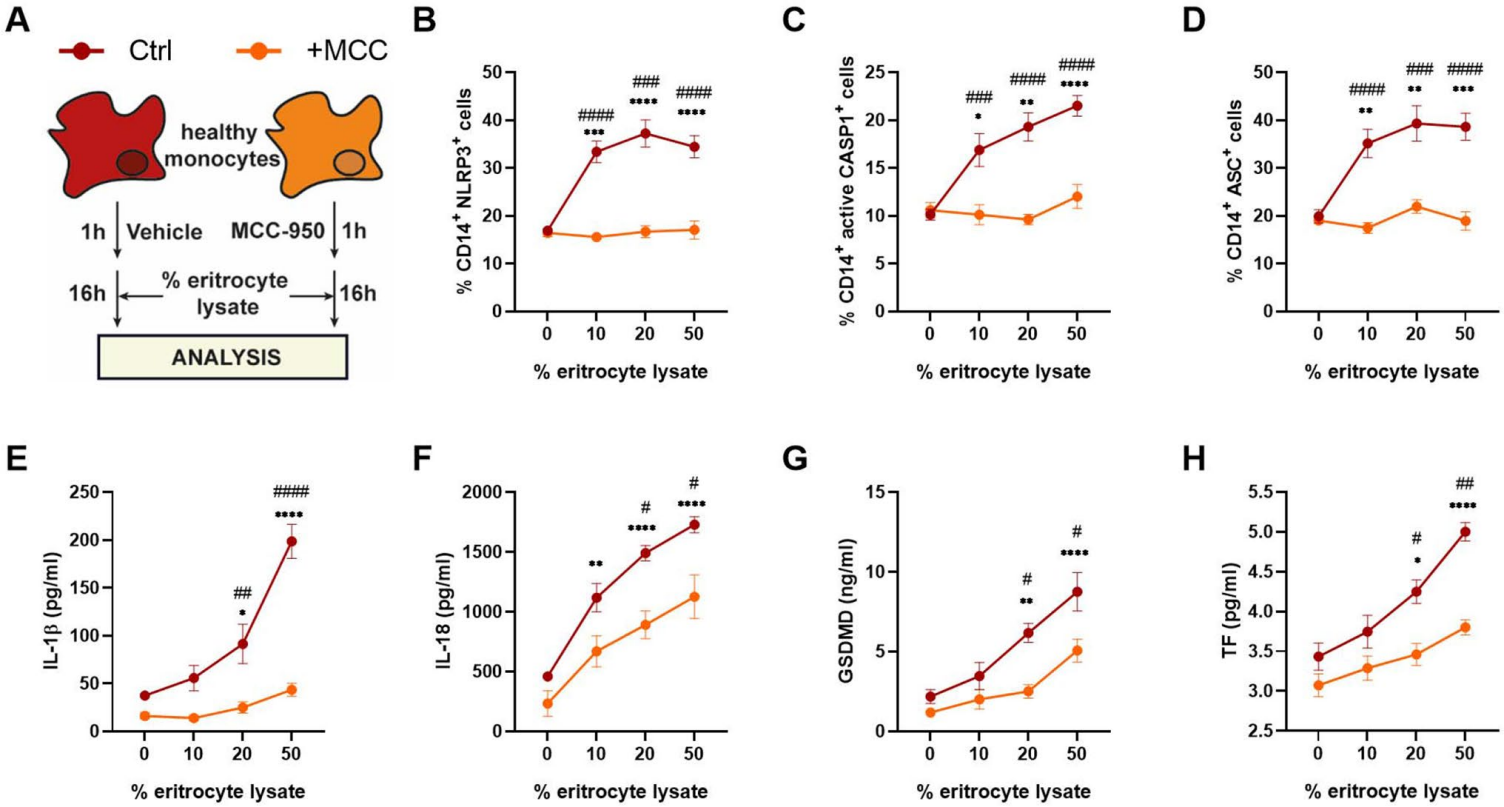
Erythrocyte lysate activates NLRP3 inflammasome. **A** Schematic representation of ex vivo experiments. **B–D** NLRP3, (**B**), ASC (**C**), and active CASP1 (**C**), expression in monocytes from CS (*n* = 4) and patients with aSAH (*n* = 5) determined by flow cytometry. **E–H** IL-1β (**E**), IL-18 (**F**), GSDMD (**G**), and TF (**H**) protein concentration determined by CBA or ELISA in supernatants from healthy volunteers (*n* = 6) stimulated with various concentrations of erythrocyte lysate under control conditions (Ctrl, red lines) or with MCC-950 treatment (+ MCC, orange lines). Comparisons between groups were performed by two-way ANOVA with Bonferroni’s multiple comparison. Mean ± SEM is shown. *P < 0.05, **P < 0.01, ***P < 0.001, ****P < 0.0001 versus Ctrl, 0% erythrocyte lysate group. ^#^*P* < 0.05, ^##^*P* < 0.01, ^###^*P* < 0.001, ^####^*P* < 0.0001 versus its paired erythrocyte lysate group treated with MCC-950

Finally, we explored the possible mechanisms underlying inflammasome activation in aSAH patients. Erythrocyte lysis and release of soluble factors into the subarachnoid space have been suspected to be potential drivers of neurological damage during aSAH [37, 38]. Nonetheless, these soluble factors resulting from hemolysis could cross the blood–brain barrier [39], which might promote NLRP3 activation in circulating monocytes. Indeed, the oxidized hemoglobin (OxyHb) has been recently reported to activate the NLRP3 inflammasome [40]. Therefore, we evaluated the potential effect of erythrolysis occurring after the aSAH event in terms of triggering NLRP3 inflammasome activation using in vitro models. Briefly, we stimulated monocytes from healthy volunteers with various concentrations of the corresponding autologous erythrocyte lysate in the presence or absence of MCC-950 (Fig. 5A). We found that NLRP3, ASC, and active-CASP-1 levels increased after erythrocyte lysate stimulation (Fig. 5B−D). Similarly, a supernatant analysis showed that erythrocyte lysate stimulation induced the release of IL-1β, IL-18, GSDMD, and TF in a dose-dependent manner (Fig. 5E−H). Interestingly, these effects were suppressed when cells were concomitantly treated with MCC-950 (Fig. 2B−H). These data show that erythrocyte lysate effectively activates the NLRP3 inflammasome.

## Discussion

**Fig. 6:**
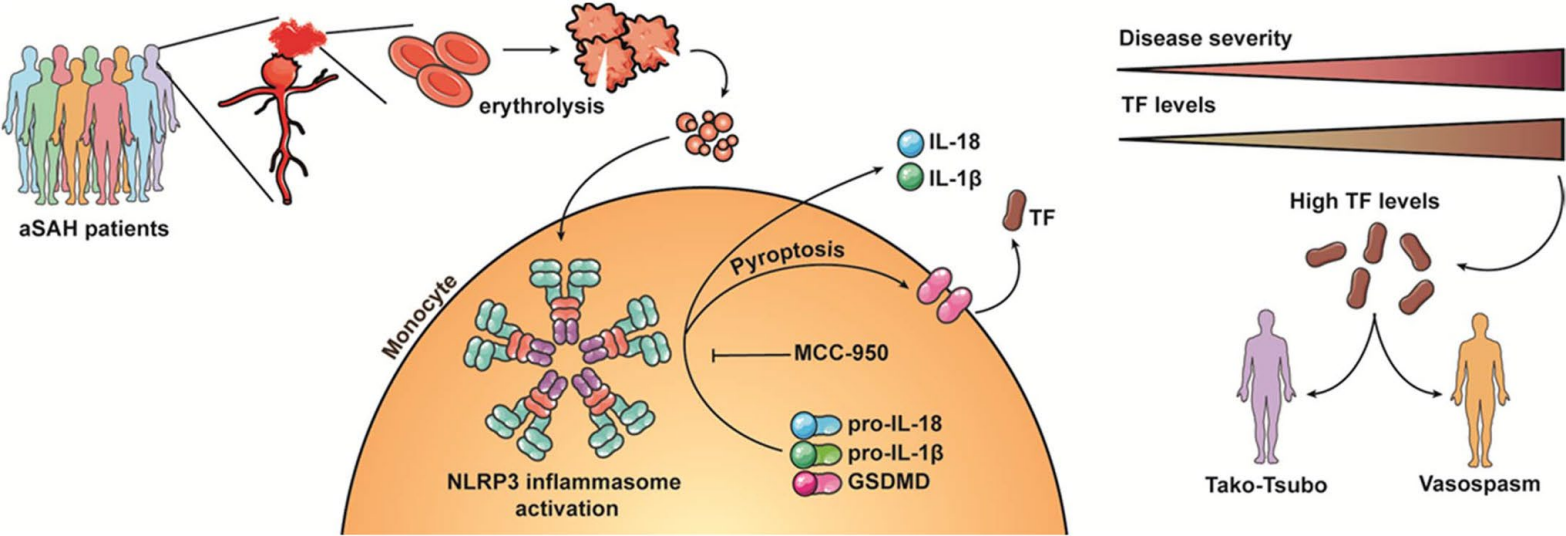
Schematic representation of main conclusions. aSAH is a devastating pathology with numerous complications that compromise patient clinical outcome. We propose that erythrolysis occurring after aSAH event promotes NLRP3 activation, which leads to the maturation and release of pro-inflammatory cytokines and also to pyroptosis-mediated TF release. TF levels were associated with a SAH severity and with clinical outcome after aSAH and were higher in patients who develop CVS and TCM, suggesting a potential role of TF as a prognosis biomarker. In this context, we demonstrated that MCC-950 NLRP3-inhibitor effectively blocks TF release from monocytes, highlighting emerging inflammasome inhibitors as a potential therapy for aSAH

Aneurysmal subarachnoid hemorrhage (aSAH) is an uncommon and severe subtype of stroke leading to the acute inflammation and secondary delayed inflammatory responses inducing morbidity and mortality. The critical role of inflammatory states as the regulator of a plethora of diseases is widely recognized, emerging as a promising area of research for potential therapeutics. Thereby, the inflammatory mechanisms must be better understood in order to efficiently manage unregulated inflammation, improving patient outcomes following aSAH and favoring the progress for clinical translation [41]. In the current study, we provide evidence of inflammasome overactivation in aSAH patients, launching pyroptosis and leading to the release of proinflammatory cytokines and TF. In fact, TF levels were associated with clinical outcome after aSAH and were higher in patients who develop CVS and TCM, suggesting a potential role of TF as a biomarker. In this context, we demonstrated that MCC-950 NLRP3-inhibitor effectively blocks inflammatory cytokines and TF release from monocytes, highlighting emerging inflammasome inhibitors as a potential therapy for aSAH. In this line, we also identified erythrolysis occurring after aSAH event as the possible mechanism underlying NLRP3 activation (Fig. 6).

Although the NLRP3 inflammasome is the master regulator of sterile inflammation [42], it has been also related with coagulation and thrombosis [43, 44]. Interestingly, NLRP3-dependent TF-release has been associated with several cardiovascular diseases including hypertension, ischemic cardiomyopathy, atherosclerosis, acute myocardial infarction, and acute coronary syndromes [45–47]. Indeed, we have found a relation between TF serum levels at admission and aSAH disease severity using the standard clinical scores. More importantly, our study indicated that serum TF might also have a potential predictive value not only for CVS but also for TCM and overall clinical outcome. In agreement, cerebrospinal fluid (CSF) membrane-bound TF has been previously suggested as indicator of tissue injury and predictor of CVS after subarachnoid hemorrhage [29, 31, 32]. Even more, serum is a much more accessible biological fluid than CSF is, rising TF value as a prognosis biomarker. Nevertheless, our TF serum data are not associated with other local aSAH complications. In contrast, recent study reported that NLRP3 activity is increased in patients with delayed neurological injury or hydrocephalus by elevated caspase 1 in CSF [31]. This discrepancy might be due to the differences in biological samples. On the other side, TF serum levels at admission day are associated with TCM in aSAH patients. However, from our data, we cannot elucidate a causal relationship between TF levels and TCM since this complication appears early in the beginning of SAH course. These results entail that aSAH patients present a systemic rather than local inflammation.

Collectively, our data indicate the systemic inflammation is regulated by NLRP3 inflammasome, as the IL-1β and IL-18 inflammatory cytokines are increased in serum from patients. Large evidence has focused in local inflammation in aSAH events either in patients and animal models; however, few studies explore the systemic inflammation. In this line, a recent systematic review highlighted the need for investigations that explore the systemic inflammatory response to contribute to the identification of biomarkers related to patient outcomes and recovery over time. So far, the inflammation state is well accepted to play a pro-coagulation role in different diseases [48]. In this sense, our results suggest NLRP3 might induce the coagulation activity by the release of TF. These evidences are in line with our proposal about the NLRP3 inflammasome activation, which might contribute to enhance the TF circulating levels increasing the coagulation activity rising the CVS after SAH event.

In particular, TF data exhibited an association with poor prognosis at the different time points during hospital stay. However, the inflammatory markers were not associated with the clinical outcomes at any time point. Indeed, there is no change in TF or inflammatory cytokine levels over the time; although there was a slight decrease in NLRP3 expressing monocytes without reaching statistical significance. Beyond its potential role as clinical biomarker, NLRP3-dependent TF release might be determinant in aSAH pathophysiology. In this line, we demonstrated that NLRP3 inhibition with MCC-950 effectively suppresses TF release from aSAH monocytes, supporting the role of emerging NLRP3 inhibitors (NLRP3i) in aSAH treatment. Indeed, targeting NLRP3 has already been explored in SAH animal models. For instance, MCC-950 attenuated early brain injury and cerebral vasospasm after experimental SAH in animal models [49, 50]. Besides, a plethora of other molecules of uneven origin rather than NLRP3i, such as melatonin [10, 11], resolvin D1 [12], the HSP90 inhibitor 17-AAG [13], the saponin dioscin [14], also plant-derived antioxidants such as resveratrol, pterostilbene, and luteolin [15–17], caspase inhibitors [30, 51, 52], and commercialized drugs from ansiolitics [18–20] to antibiotics [21], have demonstrated to alleviate neurological deficits and improve neurobehavioral outcomes in SAH animal models by targeting, from diverse pathways, NLRP3 inflammasome axis.

There is large evidence showing that NLRP3 inflammasome is essential in response to a sterile inflammatory reaction to DAMPs [25, 53]. Interestingly, aSAH pathophysiology has been associated with oxidative stress and cell apoptosis, both processes related with massive DAMPs release [52, 54]. So far, recent data mentioned that, after aSAH event, the red blood cells are degraded in the subarachnoid space promoting oxidative stress and activating inflammatory cascades [55, 56], increasing the inflammatory cytokines [41, 57, 58]. Here, we propose that erythrolysis could be the triggering factor underlying NLRP3 activation in aSAH monocytes using an in vitro model. In agreement, erythrolysis has been related with brain injury and neuroinflammation after intracerebral hemorrhage [11, 59]. Although OxyHb could be responsible for the initiation of inflammasome activation in aSAH [40], the erythrocyte lysate contains numerous substances other than OxyHb, and there are other diverse mechanisms that could activate NLRP3 inflammasome. For instance, the lysosomal membrane may be damaged after SAH, leading to the release of cathepsin B/D which induces inflammasome activation and apoptosis [60, 61]. In addition, Ca^2+^ ions from lysosomal rupture could also regulate NLRP3 inflammasome activation in SAH through the TGFbeta Activated Kinase 1/Jun N-terminal kinase (TAK1/JNK) pathway [62–64]; also, NEK7 an essential mediator of NLRP3 activation downstream of potassium efflux [65] has been reported to be implicated in neuroinflammation after SAH in mice [66]. Besides, extracellular accumulation of adenosine triphosphate (ATP) may also activate the NLRP3 inflammasome axis inducing neuroinflammation after SAH [67]. In fact, P2X7R blockade has been shown to prevent neuroinflammation after intracerebral hemorrhage in rats [68, 69].

Nevertheless, our study has several limitations, which we recognize. First, this is a unicenter study including a limited cohort so a validation cohort is needed to further confirm these results. Second, although our study demonstrates an effect of erythrolysis in NLRP3 inflammasome activation, it does not provide information about the identification and characterization or erythrocyte lysate. Third, while the monocytes are the main TF producers, it is predictable that other cell groups also contribute to its serum levels. Fourth, in this observational study, patients were treated according to conventional clinical practice depending on disease progression and complications so the non-randomization does not allow us to infer the effect of aSAH treatment on NLRP3 inflammasome activity or the progression of complications.

In summary, this study supports overactivation of NLRP3 inflammasome in human samples, opening a range of possibilities both in the prognosis and therapeutic management of this disease. Our data may reveal that NLRP3 inflammasome activation in aSAH is systemic rather than local, resulting in high levels of proinflammatory cytokines and procoagulatory TF in plasma of patients with aSAH. Moreover, although further research is needed, we observed that TF serum concentration might be a useful biomarker not only for potentially predicting aSAH clinical outcome, but also for CVS and TCM prognosis. The standardization of TF detection could bring an accessible tool for aSAH patient classification, contributing to the field of personalized medicine. However, further studies are needed to elucidate the role of TF as a prognostic biomarker. Finally, our findings suggested erythrocyte breakdown products as a triggering factor of NLRP3 activation.

## Supplementary Information

Below is the link to the electronic supplementary material.

Supplementary file1 (DOCX 330 KB)

## Data Availability

Anonymized data will be shared by request from any qualified investigator. The data that support the findings of this study are available from the corresponding author upon reasonable request. Individual level participant data will not be made available to others due to privacy concerns.

## References

[1] Etminan N, Chang HS, Hackenberg K, de Rooij NK, Vergouwen MDI, Rinkel GJE (2019). Worldwide incidence of aneurysmal subarachnoid hemorrhage according to region, time period, blood pressure, and smoking prevalence in the population: a systematic review and meta-analysis. JAMA Neurol.

[2] Macdonald RL, Schweizer TA (2017). Spontaneous subarachnoid haemorrhage. Lancet.

[3] Cahill J, Calvert JW, Zhang JH (2006). Mechanisms of early brain injury after subarachnoid hemorrhage. J Cereb Blood Flow Metab.

[4] Kusaka G, Ishikawa M, Nanda A, Granger DN, Zhang JH (2004). Signaling pathways for early brain injury after subarachnoid hemorrhage. J Cereb Blood Flow Metab.

[5] Voldby B, Enevoldsen EM (1982). Intracranial pressure changes following aneurysm rupture. Part 1: clinical and angiographic correlations. J Neurosurg.

[6] Hijdra A, Braakman R, van Gijn J, Vermeulen M, van Crevel H (1987). Aneurysmal subarachnoid hemorrhage. Complications and outcome in a hospital population. Stroke.

[7] Takase H, Chou SH, Hamanaka G, Ohtomo R, Islam MR, Lee JW (2020). Soluble vascular endothelial-cadherin in CSF after subarachnoid hemorrhage. Neurology.

[8] Boluijt J, Meijers JC, Rinkel GJ, Vergouwen MD (2015). Hemostasis and fibrinolysis in delayed cerebral ischemia after aneurysmal subarachnoid hemorrhage: a systematic review. J Cereb Blood Flow Metab.

[9] Dumont AS, Dumont RJ, Chow MM, Lin CL, Calisaneller T, Ley KF (2003). Cerebral vasospasm after subarachnoid hemorrhage: putative role of inflammation. Neurosurgery.

[10] Ersahin M, Toklu HZ, Cetinel S, Yuksel M, Yegen BC, Sener G (2009). Melatonin reduces experimental subarachnoid hemorrhage-induced oxidative brain damage and neurological symptoms. J Pineal Res.

[11] Dong Y, Fan C, Hu W, Jiang S, Ma Z, Yan X (2016). Melatonin attenuated early brain injury induced by subarachnoid hemorrhage via regulating NLRP3 inflammasome and apoptosis signaling. J Pineal Res.

[12] Wei C, Guo S, Liu W, Jin F, Wei B, Fan H (2020). Resolvin D1 ameliorates inflammation-mediated blood-brain barrier disruption after subarachnoid hemorrhage in rats by modulating A20 and NLRP3 inflammasome. Front Pharmacol.

[13] Zuo Y, Wang J, Liao F, Yan X, Li J, Huang L (2018). Inhibition of heat shock protein 90 by 17-AAG reduces inflammation via P2X7 receptor/NLRP3 inflammasome pathway and increases neurogenesis after subarachnoid hemorrhage in mice. Front Mol Neurosci.

[14] Zhang XS, Lu Y, Li W, Tao T, Wang WH, Gao S (2021). Cerebroprotection by dioscin after experimental subarachnoid haemorrhage via inhibiting NLRP3 inflammasome through SIRT1-dependent pathway. Br J Pharmacol.

[15] Zhang X, Wu Q, Zhang Q, Lu Y, Liu J, Li W (2017). Resveratrol attenuates early brain injury after experimental subarachnoid hemorrhage via inhibition of NLRP3 inflammasome activation. Front Neurosci.

[16] Liu H, Zhao L, Yue L, Wang B, Li X, Guo H (2017). Pterostilbene attenuates early brain injury following subarachnoid hemorrhage via inhibition of the NLRP3 inflammasome and Nox2-related oxidative stress. Mol Neurobiol.

[17] Zhang ZH, Liu JQ, Hu CD, Zhao XT, Qin FY, Zhuang Z (2021). Luteolin confers cerebroprotection after subarachnoid hemorrhage by suppression of NLPR3 inflammasome activation through Nrf2-dependent pathway. Oxid Med Cell Longev.

[18] Liu FY, Cai J, Wang C, Ruan W, Guan GP, Pan HZ (2018). Fluoxetine attenuates neuroinflammation in early brain injury after subarachnoid hemorrhage: a possible role for the regulation of TLR4/MyD88/NF-kappaB signaling pathway. J Neuroinflammation.

[19] Yin D, Zhou S, Xu X, Gao W, Li F, Ma Y (2018). Dexmedetomidine attenuated early brain injury in rats with subarachnoid haemorrhage by suppressing the inflammatory response: The TLR4/NF-kappaB pathway and the NLRP3 inflammasome may be involved in the mechanism. Brain Res.

[20] Li JR, Xu HZ, Nie S, Peng YC, Fan LF, Wang ZJ (2017). Fluoxetine-enhanced autophagy ameliorates early brain injury via inhibition of NLRP3 inflammasome activation following subrachnoid hemorrhage in rats. J Neuroinflammation.

[21] Li J, Chen J, Mo H, Chen J, Qian C, Yan F (2016). Minocycline protects against NLRP3 inflammasome-induced inflammation and P53-associated apoptosis in early brain injury after subarachnoid hemorrhage. Mol Neurobiol.

[22] Bai R, Lang Y, Shao J, Deng Y, Refuhati R, Cui L (2021). The role of NLRP3 inflammasome in cerebrovascular diseases pathology and possible therapeutic targets. ASN Neuro.

[23] Youm YH, Grant RW, McCabe LR, Albarado DC, Nguyen KY, Ravussin A (2013). Canonical Nlrp3 inflammasome links systemic low-grade inflammation to functional decline in aging. Cell Metab.

[24] Lamkanfi M, Dixit VM (2014). Mechanisms and functions of inflammasomes. Cell.

[25] Schroder K, Tschopp J (2010). The inflammasomes. Cell.

[26] Shi J, Zhao Y, Wang K, Shi X, Wang Y, Huang H (2015). Cleavage of GSDMD by inflammatory caspases determines pyroptotic cell death. Nature.

[27] Grover SP, Mackman N (2020). Tissue factor in atherosclerosis and atherothrombosis. Atherosclerosis.

[28] Wu C, Lu W, Zhang Y, Zhang G, Shi X, Hisada Y (2019). Inflammasome activation triggers blood clotting and host death through pyroptosis. Immunity.

[29] Golanov EV, Bovshik EI, Wong KK, Pautler RG, Foster CH, Federley RG (2018). Subarachnoid hemorrhage - induced block of cerebrospinal fluid flow: role of brain coagulation factor III (tissue factor). J Cereb Blood Flow Metab.

[30] Fang Y, Wang X, Lu J, Shi H, Huang L, Shao A (2022). Inhibition of caspase-1-mediated inflammasome activation reduced blood coagulation in cerebrospinal fluid after subarachnoid haemorrhage. EBioMedicine.

[31] Hirashima Y, Endo S, Nakamura S, Kurimoto M, Takaku A (2001). Cerebrospinal fluid membrane-bound tissue factor and myelin basic protein in the course of vasospasm after subarachnoid hemorrhage. Neurol Res.

[32] Hirashima Y, Nakamura S, Suzuki M, Kurimoto M, Endo S, Ogawa A (1997). Cerebrospinal fluid tissue factor and thrombin-antithrombin III complex as indicators of tissue injury after subarachnoid hemorrhage. Stroke.

[33] Steiner T, Juvela S, Unterberg A, Jung C, Forsting M, Rinkel G (2013). European Stroke Organization guidelines for the management of intracranial aneurysms and subarachnoid haemorrhage. Cerebrovasc Dis.

[34] Burns SK, Brewer KJ, Jenkins C, Miller S (2018). Aneurysmal subarachnoid hemorrhage and vasospasm. AACN Adv Crit Care.

[35] Juvela S, Siironen J (2006). D-dimer as an independent predictor for poor outcome after aneurysmal subarachnoid hemorrhage. Stroke.

[36] Liesirova K, Abela E, Pilgrim T, Bickel L, Meinel T, Meisterernst J (2018). Baseline Troponin T level in stroke and its association with stress cardiomyopathy. PLoS ONE.

[37] Kolias AG, Sen J, Belli A (2009). Pathogenesis of cerebral vasospasm following aneurysmal subarachnoid hemorrhage: putative mechanisms and novel approaches. J Neurosci Res.

[38] Macdonald RL, Weir BK (1991). A review of hemoglobin and the pathogenesis of cerebral vasospasm. Stroke.

[39] Butt OI, Buehler PW, D'Agnillo F (2011). Blood-brain barrier disruption and oxidative stress in guinea pig after systemic exposure to modified cell-free hemoglobin. Am J Pathol.

[40] Nyakundi BB, Toth A, Balogh E, Nagy B, Erdei J, Ryffel B (2019). Oxidized hemoglobin forms contribute to NLRP3 inflammasome-driven IL-1beta production upon intravascular hemolysis. Biochim Biophys Acta Mol Basis Dis.

[41] Devlin P, Ishrat T, Stanfill AG. A systematic review of inflammatory cytokine changes following aneurysmal subarachnoid hemorrhage in animal models and humans. Transl Stroke Res. 2022. 10.1007/s12975-022-01001-y. 10.1007/s12975-022-01001-y 35260989

[42] Abderrazak A, Syrovets T, Couchie D, El Hadri K, Friguet B, Simmet T (2015). NLRP3 inflammasome: from a danger signal sensor to a regulatory node of oxidative stress and inflammatory diseases. Redox Biol.

[43] Bogdanov VY, Osterud B (2010). Cardiovascular complications of diabetes mellitus: the Tissue Factor perspective. Thromb Res.

[44] Owens AP, Byrnes JR, Mackman N (2014). Hyperlipidemia, tissue factor, coagulation, and simvastatin. Trends Cardiovasc Med.

[45] Diaz-Garcia E, Garcia-Tovar S, Alfaro E, Jaureguizar A, Casitas R, Sanchez-Sanchez B (2022). Inflammasome activation: a keystone of proinflammatory response in obstructive sleep apnea. Am J Respir Crit Care Med.

[46] Morange PE, Blankenberg S, Alessi MC, Bickel C, Rupprecht HJ, Schnabel R (2007). Prognostic value of plasma tissue factor and tissue factor pathway inhibitor for cardiovascular death in patients with coronary artery disease: the AtheroGene study. J Thromb Haemost.

[47] Suefuji H, Ogawa H, Yasue H, Kaikita K, Soejima H, Motoyama T (1997). Increased plasma tissue factor levels in acute myocardial infarction. Am Heart J.

[48] Foley JH, Conway EM (2016). Cross talk pathways between coagulation and inflammation. Circ Res.

[49] Luo Y, Lu J, Ruan W, Guo X, Chen S (2019). MCC950 attenuated early brain injury by suppressing NLRP3 inflammasome after experimental SAH in rats. Brain Res Bull.

[50] Dodd WS, Noda I, Martinez M, Hosaka K, Hoh BL (2021). NLRP3 inhibition attenuates early brain injury and delayed cerebral vasospasm after subarachnoid hemorrhage. J Neuroinflammation.

[51] Iseda K, Ono S, Onoda K, Satoh M, Manabe H, Nishiguchi M (2007). Antivasospastic and antiinflammatory effects of caspase inhibitor in experimental subarachnoid hemorrhage. J Neurosurg.

[52] Zhou C, Yamaguchi M, Kusaka G, Schonholz C, Nanda A, Zhang JH (2004). Caspase inhibitors prevent endothelial apoptosis and cerebral vasospasm in dog model of experimental subarachnoid hemorrhage. J Cereb Blood Flow Metab.

[53] Schroder K, Zhou R, Tschopp J (2010). The NLRP3 inflammasome: a sensor for metabolic danger?. Science.

[54] Vecchione C, Frati A, Di Pardo A, Cifelli G, Carnevale D, Gentile MT (2009). Tumor necrosis factor-alpha mediates hemolysis-induced vasoconstriction and the cerebral vasospasm evoked by subarachnoid hemorrhage. Hypertension.

[55] Pan P, Xu L, Zhang H, Liu Y, Lu X, Chen G (2020). A review of hematoma components clearance mechanism after subarachnoid hemorrhage. Front Neurosci.

[56] Ma B, Day JP, Phillips H, Slootsky B, Tolosano E, Dore S (2016). Deletion of the hemopexin or heme oxygenase-2 gene aggravates brain injury following stroma-free hemoglobin-induced intracerebral hemorrhage. J Neuroinflammation.

[57] Ganz T. Macrophages and Iron Metabolism. Microbiol Spectr. 2016;4(5):MCHD-0037-2016. 10.1128/microbiolspec.MCHD-0037-2016 27763254

[58] Blackburn SL, Kumar PT, McBride D, Zeineddine HA, Leclerc J, Choi HA (2018). Unique contribution of haptoglobin and haptoglobin genotype in aneurysmal subarachnoid hemorrhage. Front Physiol.

[59] Wang M, Xia F, Wan S, Hua Y, Keep RF, Xi G (2021). Role of complement component 3 in early erythrolysis in the hematoma after experimental intracerebral hemorrhage. Stroke.

[60] Tang T, Lang X, Xu C, Wang X, Gong T, Yang Y (2017). CLICs-dependent chloride efflux is an essential and proximal upstream event for NLRP3 inflammasome activation. Nat Commun.

[61] Hornung V, Bauernfeind F, Halle A, Samstad EO, Kono H, Rock KL (2008). Silica crystals and aluminum salts activate the NALP3 inflammasome through phagosomal destabilization. Nat Immunol.

[62] Okada M, Matsuzawa A, Yoshimura A, Ichijo H (2014). The lysosome rupture-activated TAK1-JNK pathway regulates NLRP3 inflammasome activation. J Biol Chem.

[63] Zhou K, Enkhjargal B, Xie Z, Sun C, Wu L, Malaguit J (2018). Dihydrolipoic acid inhibits lysosomal rupture and NLRP3 through lysosome-associated membrane protein-1/calcium/calmodulin-dependent protein kinase II/TAK1 pathways after subarachnoid hemorrhage in rat. Stroke.

[64] Xu P, Tao C, Zhu Y, Wang G, Kong L, Li W (2021). TAK1 mediates neuronal pyroptosis in early brain injury after subarachnoid hemorrhage. J Neuroinflammation.

[65] He Y, Zeng MY, Yang D, Motro B, Nunez G (2016). NEK7 is an essential mediator of NLRP3 activation downstream of potassium efflux. Nature.

[66] Li G, Dong Y, Liu D, Zou Z, Hao G, Gao X (2020). NEK7 coordinates rapid neuroinflammation after subarachnoid hemorrhage in mice. Front Neurol.

[67] Chen S, Ma Q, Krafft PR, Hu Q, Rolland W, Sherchan P (2013). P2X7R/cryopyrin inflammasome axis inhibition reduces neuroinflammation after SAH. Neurobiol Dis.

[68] Zhao H, Pan P, Yang Y, Ge H, Chen W, Qu J (2017). Endogenous hydrogen sulphide attenuates NLRP3 inflammasome-mediated neuroinflammation by suppressing the P2X7 receptor after intracerebral haemorrhage in rats. J Neuroinflammation.

[69] Feng L, Chen Y, Ding R, Fu Z, Yang S, Deng X (2015). P2X7R blockade prevents NLRP3 inflammasome activation and brain injury in a rat model of intracerebral hemorrhage: involvement of peroxynitrite. J Neuroinflammation.

