# Supplementary material for: NLRP3 Inflammasome Overactivation in Patients with Aneurysmal Subarachnoid Hemorrhage
Source: Transl Stroke Res. 2022 Jul 11;14(3):334–46. doi: 10.1007/s12975-022-01064-x (PMC10160181; doi:10.1007/s12975-022-01064-x)
Supplement: Supplementary file 1 — Supplementary file1 (DOCX 330 KB) [file 12975_2022_1064_MOESM1_ESM.docx]

**SUPPLEMENTAL DATA**

**NLRP3 inflammasome overactivation in patients with aneurysmal subarachnoid haemorrhage**

Elena Díaz-García^1,2,*^, Kapil Nanwani-Nanwani^3,*^,Sara García-Tovar^1^, Enrique Alfaro^1^, Eduardo López-Collazo^4^, Manuel Quintana-Díaz^3,5^, Francisco García-Rio^1,2,5^, Carolina Cubillos-Zapata^1,2^

^1^Respiratory Diseases Group, Respiratory Service, La Paz University Hospital, IdiPAZ, Madrid, Spain; ^2^Biomedical Research Networking Center on Respiratory Diseases (CIBERES), Madrid, Spain; ^3^Department of Intensive Medicine, La Paz University Hospital, Madrid, Spain; ^4^The Innate Immune Response Group, La Paz University Hospital, IdiPAZ, Madrid, Spain; and ^5^Faculty of Medicine, Autonomous University of Madrid, Madrid, Spain.

**SUPPLEMENTAL TABLES**

**Table e1.** Flow cytometry anti-human antibodies used in the study.

| Target | Fluorochrome | Manufacturer | Reference |
| --- | --- | --- | --- |
| CD14 | APC | Inmunostep | 14A-100T |
| NLRP3 | PE | MACS Miltenyi Biotec | 130-111-209 |
| ASC | PE | Biolegend | 653904 |

NLRP3, nucleotide-binding oligomerization domain-like receptor 3. ASC, adaptor molecule apoptosis-associated speck-like protein. PE, phycoerythrin. APC, allophycocyanin-

**Table e2** ELISA kits used in the study

| Target | Detection limit | Manufacturer | Reference | |
| --- | --- | --- | --- | --- |
| IL-18 | 31.25pg/ml | CUSABIO technology | | CSB-E07450h |
| GSDMD | 0.312ng/mL | MyBioSource | | MBS2705515 |
| TF | 3.12pg/mL | CUSABIO technology | | CSB-E07913h |

IL, interleukin. GSDMD, Gasdermin D. TF, tissue factor**.**

**Table e3.** qPCR Primer sequences used in the study

| NLRP3 | Forward primer | TGCCCGTCTGGGTGAGA |
| --- | --- | --- |
|  | Reverse primer | CCGGTGCTCCTTGATGAGA |
| ASC | Forward primer | AACCCAAGCAAGATGCGGAAG |
|  | Reverse primer | TTAGGGCCTGGAGGAGCAAG |
| CASP-1 | Forward primer | GGAAACAAAAGTCGGCAGAG |
|  | Reverse primer | ACGCTGTACCCCAGATTTTG |
| IL-1β | Forward primer | GGATATGGAGCAACAAGTGG |
|  | Reverse primer | ATGTACCAGTTGGGGAACTG |
| IL-18 | Forward primer | TTCCAGTTACGTTCCTTCGATCA |
|  | Reverse primer | CTGGCACCTGCTGCAGTCTA |
| GSDMD | Forward primer | GTGTGTCAACCTGTCTATCAAGG |
|  | Reverse primer | CATGGCATCGTAGAAGTGGAAG |
| TF | Forward primer | CCCAAACCCGTCAATCAAGTC |
|  | Reverse primer | CCAAGTACGTCTGCTTCACAT |
| 18S | Forward primer | CGGCGACGACCCATTCGAAC |
|  | Reverse primer | GAATCGAACCCTGATTCCCCGTC |

NLRP3, nucleotide-binding oligomerization domain-like receptor 3. ASC, adaptor molecule apoptosis-associated speck-like protein. CASP-1, caspase-1. IL, interleukin. GSDMD, Gasdermin D. TF, tissue factor**.**

**Table e4.** NLRP3 inflammasome components and products levels in normal controls and patients’ monocytes.

|  | NC | aSAH | p-value |
| --- | --- | --- | --- |
| %CD14^+^ NLRP3^+^ | 14.75±1.97 (14) | 26.63±2.97 (28) | 0.0061 |
| %CD14^+^ ASC^+^ | 16.70±2.14 (14) | 35.26±3.86 (28) | 0.0018 |
| %CD14^+^ active CASP1^+^ | 6.02±1.55(14) | 15.10±3.11 (28) | 0.0011 |
| NLRP3/β-actin | 0.77±0.04(3) | 1.78±0.32(6) | 0.0238 |
| Cleaved-CASP1/β-actin | 0.41±0.10(3) | 1.09±0.15(6) | 0.0476 |
| IL-1β (pg/ml) | 2.96±0.24 (14) | 5.60±0.32 (28) | 0.0261 |
| IL-18 (pg/ml) | 1.59±0.46 (14) | 8.03±2.81 (28) | 0.0022 |
| GSDMD (pg/ml) | 1.04±0.04 (14) | 3.66±0.75 (28) | 0.0393 |
| TF (pg/ml) | 10.11±1.65 (14) | 25.25±4.85 (28) | 0.0002 |

Data are presented as Mean ± SEM (n) is represented, unpaired t-tests p-values are shown. NLRP3, nucleotide-binding oligomerization domain-like receptor 3. ASC, adaptor molecule apoptosis-associated speck-like protein. CASP-1, caspase-1. IL, interleukin. GSDMD, Gasdermin D. TF, tissue factor**.**

**Table e5.** Inflammasome soluble products correlation with clinical severity indicators

| Target | IL-1β | | | IL-18 | | | GSDMD | | |
| --- | --- | --- | --- | --- | --- | --- | --- | --- | --- |
|  | rho | p-value | n | rho | p-value | n | rho | p-value | n |
| Apache II | -0.101 | 0.609 | 28 | 0.420 | 0.029 | 28 | -0.023 | 0.885 | 28 |
| SOFA | -0.025 | 0.868 | 28 | 0.284 | 0.159 | 28 | 0.093 | 0.645 | 28 |
| WFNS | -0.186 | 0.343 | 28 | 0.249 | 0.210 | 28 | 0.052 | 0.792 | 28 |
| GCS | 0.189 | 0.335 | 28 | -0.264 | 0.184 | 28 | -0.028 | 0.887 | 28 |
| GCS-E | 0.151 | 0.442 | 28 | -0.209 | 0.295 | 28 | 0.086 | 0.663 | 28 |
| GCS-V | 0.206 | 0.293 | 28 | -0.359 | 0.066 | 28 | -0.188 | 0.338 | 28 |
| GCS-M | 0.161 | 0.412 | 28 | 0.287 | 0.147 | 28 | 0.040 | 0.840 | 28 |

IL, interleukin. GSDMD, Gasdermin D. Apache II, Acute Physiology and Chronic Health Disease Classification System II score. SOFA, Sequential Organ Failure Assessment. WFNS, World Federation of Neurological Surgeons score. GCS, Glasgow coma scale score and its components GCS-eyes score (GCS-E), GCS-verbal score (GCS-V) and GCS-motor score (GCS-M). Spearman’s correlation coefficients (rho) and p-values are shown.

**Table e6.** Serum tissue factor levels in patients with good or poor outcome.

| Variable | Poor GOS-E_6m | Good GOS-E_6m | p-value |
| --- | --- | --- | --- |
| Day 1 | 31.50±8.74 (15) | 11.03±2.30 (12) | 0.0378 |
| Day 2-5 | 34.04±5.46(12) | 18.20±3.15(10) | 0.0223 |
| Day 7-10 | 40.65±7.54 (12) | 22.24±4.13 (10) | 0.0470 |

Serum tissue factor concentration data are presented as Mean ± SEM (n) is represented, unpaired t-tests p-values are shown. GOS-E_6m. Glasgow Outcome Scale Extended at 6 months after discharge from the ICU. GOS-E_6m, Poor <8, Good =8.

**Table e7.** Serum tissue factor levels in patients with and without complications.

| Variable | NO | YES | p-value |
| --- | --- | --- | --- |
| Vasospasm | 15.78±2.30 (19) | 37.11±14.43 (9) | 0.0482 |
| Acute hydrocephalus | 15.09±3.02(14) | 30.18±9.47(14) | 0.1412 |
| Chronic hydrocephalus | 17.65±3.02 (15) | 28.39±10.40 (13) | 0.3015 |
| Delayed neurological injury | 23.27±6.17 (23) | 19.69±3.83 (5) | 0.7933 |
| Tako-Tsubo | 17.29±2.22 (22) | 42.24±21.88 (6) | 0.0418 |

Serum tissue factor concentration data are presented as Mean ± SEM (n) is represented, unpaired t-tests p-values are shown.

| Variable | NC | | | aSAH | | | p-value | |
| --- | --- | --- | --- | --- | --- | --- | --- | --- |
|  | **C** | **MCC** | **p-value**  **C vs MCC** | **C** | **MCC** | **p-value**  **C vs MCC** | **C (NC vs aSAH)** | **MCC (NC vs aSAH)** |
| %CD14^+^ NLRP3^+^ (Fold induction) | 1.00±0.06 (4) | 0.81±0.17 (4) | 0.8120 | 1.75±0.23 (5) | 0.93±.0.19 (5) | 0.0060 | 0.0290 | >0.9999 |
| %CD14^+^ ASC^+^ (Fold induction) | 1.00±0.32 (4) | 0.85±0.33 (4) | >0.9999 | 2.97±0.64 (5) | 1.41±.0.25 (5) | 0.0030 | 0.0136 | 0.7614 |
| %CD14^+^ active CASP1^+^ ^+^(Fold induction) | 1.00±0.06(4) | 0.62±0.10 (4) | 0.2784 | 4.55±0.24 (5) | 2.342±.0.29 (5) | <0.0001 | <0.0001 | 0.0001 |
| IL-1β (pg/ml) | 30.04±5.99 (4) | 11.26±1.47 (4) | 0.0635 | 84.51±13.94 (5) | 54.71±.9.54 (5) | 0.0042 | 0.0034 | 0.0160 |
| IL-18 (pg/ml) | 433.59±9.24 (3) | 358.22±30.78 (3) | 0.0412 | 611.02±25.96 (6) | 559.39±.29.59(6) | 0.0471 | 0.0022 | 0.0008 |
| GSDMD (pg/ml) | 1.74±0.32 (4) | 1.20±0.11 (4) | 0.6511 | 4.11±0.61 (5) | 1.36±.0.13 (5) | 0.0011 | 0.0133 | >0.9999 |
| TF (pg/ml) | 3.22±0.32 (4) | 3.20±0.53 (4) | >0.9999 | 7.15±0.58 (5) | 6.58±.0.44(5) | 0.0020 | <0.0001 | <0.0001 |

**Table e8.** NLRP3 inflammasome components and products levels in normal controls and patients’ monocytes treated or not with MCC-950.

Data are presented as Mean ± SEM (n) is represented, Two-way ANOVA p-values are shown. NLRP3, nucleotide-binding oligomerization domain-like receptor 3. ASC, adaptor molecule apoptosis-associated speck-like protein. CASP-1, caspase-1. IL, interleukin. GSDMD, Gasdermin D. TF, tissue factor. C, control conditions. MCC, MCC-950 treatment**.**

**SUPPLEMENTAL FIGURES**

**Figure e1.** Gating strategy for cytometry analysis. Gray squares, plots corresponding to NC subjects. Brown squares, plots corresponding to aSAH subjects.

**Figure e2**. **A**, NLRP3 (expression in monocytes from in patients with aSAH over time determined by flow cytometry, (day 1 n= 28, day 2-5 n=17, and day 7-10 n=18). **B-E**, IL-1β (B), IL-18 (C), GSDMD (D) and TF (E) protein concentration in serum patients with aSAH over time determined CBA or enzyme-linked immunosorbent assay (ELISA), (day 1 n= 28, day 2-5 n=25, and day 7-10 n=24). Comparisons between groups were performed by unpaired t test. Mean ± SEM is shown. **F**, Heatmap of NLRP3 inflammasome components and end-products mRNA relative expression in aSAH and CS subjects.

**Figure e3**. **A**, Correlation between TF and D-dimer protein concentration determined by ELISA in serum from aSAH patients. **B**, Correlation between TF and troponin protein concentration determined by ELISA in serum from aSAH patients. C, Correlation between NLRP3 expression in aSAH monocytes determined by flow cytometry and TF protein concentration determined by ELISA in serum from aSAH patients. Spearman’s correlation coefficients (ρ) and p-values are shown.
